# Intracortical diffusion tensor imaging signature of microstructural changes in frontotemporal lobar degeneration

**DOI:** 10.1186/s13195-021-00914-4

**Published:** 2021-10-22

**Authors:** Mario Torso, Gerard R. Ridgway, Mark Jenkinson, Steven Chance, the Frontotemporal Lobar Degeneration Neuroimaging Initiative and the 4-Repeat Tau Neuroimaging Initiative (4RTNI)

**Affiliations:** 1 Nuffield Department of Clinical Neuroscience, University of Oxford, Oxford, UK; 2 Oxford Brain Diagnostics Limited, Oxford, UK; 3 Wellcome Centre for Integrative Neuroimaging, FMRIB, Nuffield Department of Clinical Neurosciences, University of Oxford, Oxford, UK

**Keywords:** Diffusion tensor imaging, FTLD, Minicolumn, Cortex, Microstructural, FTD, PSP, CBS, Intracortical, Cortical diffusivity

## Abstract

**Background:**

Frontotemporal lobar degeneration (FTLD) is a neuropathological construct with multiple clinical presentations, including the behavioural variant of frontotemporal dementia (bvFTD), primary progressive aphasia—both non-fluent variant (nfvPPA) and semantic variant (svPPA)—progressive supranuclear palsy (PSP) and corticobasal syndrome (CBS), characterised by the deposition of abnormal tau protein in the brain. A major challenge for treating FTLD is early diagnosis and accurate discrimination among different syndromes. The main goal here was to investigate the cortical architecture of FTLD syndromes using cortical diffusion tensor imaging (DTI) analysis and to test its power to discriminate between different clinical presentations.

**Methods:**

A total of 271 individuals were included in the study: 87 healthy subjects (HS), 31 semantic variant primary progressive aphasia (svPPA), 37 behavioural variant (bvFTD), 30 non-fluent/agrammatic variant primary progressive aphasia (nfvPPA), 47 PSP Richardson’s syndrome (PSP-RS) and 39 CBS cases. 3T MRI T1-weighted images and DTI scans were analysed to extract three cortical DTI derived measures (AngleR, PerpPD and ParlPD) and mean diffusivity (MD), as well as standard volumetric measurements. Whole brain and regional data were extracted. Linear discriminant analysis was used to assess the group discrimination capability of volumetric and DTI measures to differentiate the FTLD syndromes. In addition, in order to further investigate differential diagnosis in CBS and PSP-RS, a subgroup of subjects with autopsy confirmation in the training cohort was used to select features which were then tested in the test cohort.

Three different challenges were explored: a binary classification (controls vs all patients), a multiclass classification (HS vs bvFTD vs svPPA vs nfvPPA vs CBS vs PSP-RS) and an additional binary classification to differentiate CBS and PSP-RS using features selected in an autopsy confirmed subcohort.

**Results:**

Linear discriminant analysis revealed that PerpPD was the best feature to distinguish between controls and all patients (ACC 86%). PerpPD regional values were able to classify correctly the different FTLD syndromes with an accuracy of 85.6%. The PerpPD and volumetric values selected to differentiate CBS and PSP-RS patients showed a classification accuracy of 85.2%.

**Conclusions:**

(I) PerpPD achieved the highest classification power for differentiating healthy controls and FTLD syndromes and FTLD syndromes among themselves. **(**II) PerpPD regional values could provide an additional marker to differentiate FTD, PSP-RS and CBS.

## Background

Frontotemporal lobar degeneration (FTLD) is a neuropathological construct with multiple clinical presentations, including the behavioural variant of frontotemporal dementia (bvFTD) [1], primary progressive aphasia (PPA) (non-fluent (nfvPPA) and semantic (svPPA) variant [2]), progressive supranuclear palsy [3, 4] and corticobasal syndrome (CBS) [5], characterised by the deposition of abnormal proteins in the brain. A major challenge for treating FTLD is early diagnosis and accurate discrimination among different forms. FTLD is related to a broad spectrum of phenotypes and the current clinical criteria do not reliably predict underlying proteinopathies antemortem [6].

Neuropathologically, FTLD can be classified into different subgroups: FTLD-Tau that includes 3-repeated (Pick’s disease (PiD)) and 4-repeated tau (PSP, CBD, argyrophilic grain disease (AGD)), FTLD with TDP-43 inclusions (type A, B, C and D), FTLD with FET protein accumulation (atypical FTLD-U, basophilic inclusion body disease (BIBD), neuronal intermediate filament inclusion disease (NIFID)) and FTLD ubiquitin/proteasome system (FTLD-UPS) [7]. Other neurodegenerative conditions with diverse aetiologies may be associated with tau pathology; some of these are known as secondary tauopathies as other proteins play a central role in their pathogenesis [8]. Neuropathological diagnosis is currently based mainly on post-mortem assessment, wherein the detection of TAR DNA-binding protein 43 (TDP-43), hyperphosphorylated tau protein, and the process of distinguishing tau isoforms are the most typical methods. There are six isoforms of the microtubule-associated protein, tau, in the adult human brain, derived from exon 10 splicing, producing two major classes of tau, those with three repeats (3R tau) and those with four repeats (4R tau) in the microtubule-binding domain of tau.

While the patient is alive, neuroimaging could offer promising biomarkers and an important in-vivo diagnostic support by providing novel measures of brain degeneration. Some previous studies using MRI, PET and SPECT imaging in FTD populations showed patterns of atrophy mainly in the fronto-temporal regions [5]. Fluorodeoxyglucose positron emission tomography (FDG-PET), functional MRI and single-photon-emission CT (SPECT) likewise show disproportionate hypoperfusion and hypometabolism in these regions [9].

Other studies in CBD populations have identified patterns of grey matter loss in the basal ganglia/thalamus, frontal, parietal and temporal lobes as a CBD signature [10, 11]. PSP has been described to be usually associated with midbrain atrophy, well known in conventional structural MRI as “morning glory sign” or “Mickey Mouse sign” [12, 13]. However, these radiological patterns have shown an inconsistent range of sensitivity and specificity [13–16].

In the present work, we suggest that the cortical microstructure (as measured by DTI) could represent a potential biomarker of neurodegeneration in FTLD-related syndromes. Previous histological studies [17, 18] have shown that changes in cortical architecture, caused by neurodegenerative processes and protein deposition, produced alteration in the cortical geometrical properties in the form of minicolumn organisation. Minicolumn degeneration varies between brain regions, reflecting the typical pattern of vulnerability to tau tangle accumulation [19]. These differences between brain regions suggest that microstructural changes in cortical grey matter, with regional variation, may be sensitive for differentiating between neurodegenerative variants.

This study investigated the cortical architecture features in several FTLD syndromes (bvFTD, nfvPPA, svPPA, CBS and PSP) and a control group, using a novel diffusion tensor imaging (DTI) analysis method [20]. Cortical diffusion measures previously validated in a post-mortem cohort [21] were used to interrogate diffusion scans from individuals with various FTLD syndromes. Recent evidence has shown that the cortical diffusion measures are sensitive to cortical changes [17, 19–25] and protein deposition [26–28].

The main goals of the present study were to (i) differentiate patients from healthy control individuals, (ii) explore a diffusion measure in the cortex that could assist in differential diagnosis and (iii) investigate the differentiation of CBD from PSP in a subcohort of individuals with autopsy confirmation. This last differentiation is of particular clinical importance since accurate diagnosis is necessary for improving the understanding of underlying neuropathology. PSP and CBD present partially overlapping topographies of neurodegeneration and clinical phenotypes that can lead to frequent misdiagnosis [29, 30].

## Methods

### Participants

A total of 271 individuals were included in the present study: 87 healthy subjects (HS), 31 semantic variant primary progressive aphasia (svPPA) [2], 37 behavioural-variant of fronto-temporal dementia (bvFTD) [1], 30 non-fluent/agrammatic variant primary progressive aphasia (nfvPPA) [2], 47 progressive supranuclear palsy Richardson's syndrome (PSP-RS) [3, 4] and 39 corticobasal syndrome (CBS) [5] cases. The FTD and control groups were from the frontotemporal lobar degeneration neuroimaging initiative (FTLDNI). PSP-RS and CBS groups were from the 4-Repeat Tau Neuroimaging Initiative (4RTNI, http://4rtni-ftldni.ini.usc.edu). The imaging and clinical methods are the same for both initiatives.

FTLDNI was founded through the National Institute of Aging and started in 2010. The primary aims of FTLDNI are to identify neuroimaging modalities and methods of analysis for tracking frontotemporal lobar degeneration (FTLD) and to compare the value of neuroimaging with other biomarkers in diagnostic roles. The Principal Investigator of FTLDNI is Dr. Howard Rosen (University of California, San Francisco). The data is the result of collaborative efforts at three different sites in North America. For more information, please visit http://memory.ucsf.edu/research/studies/nifd [https://ida.loni.usc.edu/collaboration/access/appLicense.jsp]. Access to the FTLDNI data was approved by the data access committee.

All subjects underwent an extensive clinical and neuropsychological evaluation and an MRI scan. The clinical diagnoses were made according to the current criteria [1–5]. Patients with vascular, psychiatric or other neurological disorders were excluded. All subjects included in the study were collected in the same centre with a similar acquisition protocol, in order to control potential confounding effects that could be caused by introducing variable acquisition protocols.

The sample was split, in order to have 2 different cohorts: the first group was used as a “Training cohort” to train a classification model (respectively 58 HS, 21 svPPA, 15 bvFTD, 17 nfvPPA, 24 CBS and 32 PSP-RS).

The second group was used as a “Test cohort” to test the model created in the training cohort, in an independent sample (respectively 29 HS, 16 svPPA, 16 bvFTD, 13 nfvPPA, 15 CBS and 15 PSP-RS).

Although the scans were acquired using the same acquisition protocol, a difference in the repetition time (TR 8200 or 6600) between subjects was found for a large portion of the cohort and this was used as a criterion to divide the sample into the 2 cohorts (Training and Test).

A subset of 8 individuals with autopsy confirmation (3 CBD and 5 PSP) from within the training cohort, was investigated separately to find the best cortical and subcortical features to differentiate the two groups. These features were used to create a model using the remaining subjects of the training cohort and then tested on the test cohort.

### MRI data acquisition and pre-processing

MR images were acquired on a 3 Tesla Siemens Tim Trio system equipped with a 12-channel head coil at the UCSF Neuroscience Imaging Center, including the following acquisition:

Training cohort: (1) T1 MPRAGE (TR=2300 ms, TE=2.9 ms, matrix =240×256×160, slice thickness= 1 mm); (2) Diffusion sequences were acquired using the following parameters: TR/TE 8200/86 ms, b factor= 2000 s/mm^2^, isotropic voxels 2.2 mm^3^, 64 images with diffusion gradient applied in 64 non-collinear directions, 1 image with no diffusion weighting (b0).

Test cohort: (1) T1 MPRAGE (TR=2300 ms, TE=2.9 ms, matrix =240×256×160, slice thickness= 1 mm); (2) Diffusion sequences were acquired using the following parameters: TR/TE 6600/86 ms, b factor= 2000 s/mm^2^, isotropic voxels 2.2 mm^3^, 64 images with diffusion gradient applied in 64 non-collinear directions, 1 image with no diffusion weighting (b0).

The 3D T1-weighted image for each subject was segmented using the recon-all script included in Freesurfer v6.0 (http://surfer.nmr.mgh.harvard.edu/). The segmented masks were used to estimate the cortical thickness and the volumes of cortical grey matter (GM). The Brainstem Substructures tool [31] included in Freesurfer v6.0, was used for the segmentation of brainstem and four subregions (medulla oblongata, pons, superior cerebellar peduncle and midbrain). To account for head size, all volumes were normalised for total intracranial volume and expressed as fractions (fr).

All DTI images were processed using the FMRIB software library, (FSL Version 6.0.1, FMRIB, Oxford, UK, http://www.fmrib.ox.ac.uk/fsl/). Data was corrected for eddy and head motion and the diffusion tensor model at each voxel was fitted using DTIFIT. To control for the effect of head motion in DTI maps, a displacement index was calculated using an in-house script.

### Cortical diffusivity analysis

**Fig. 1:**
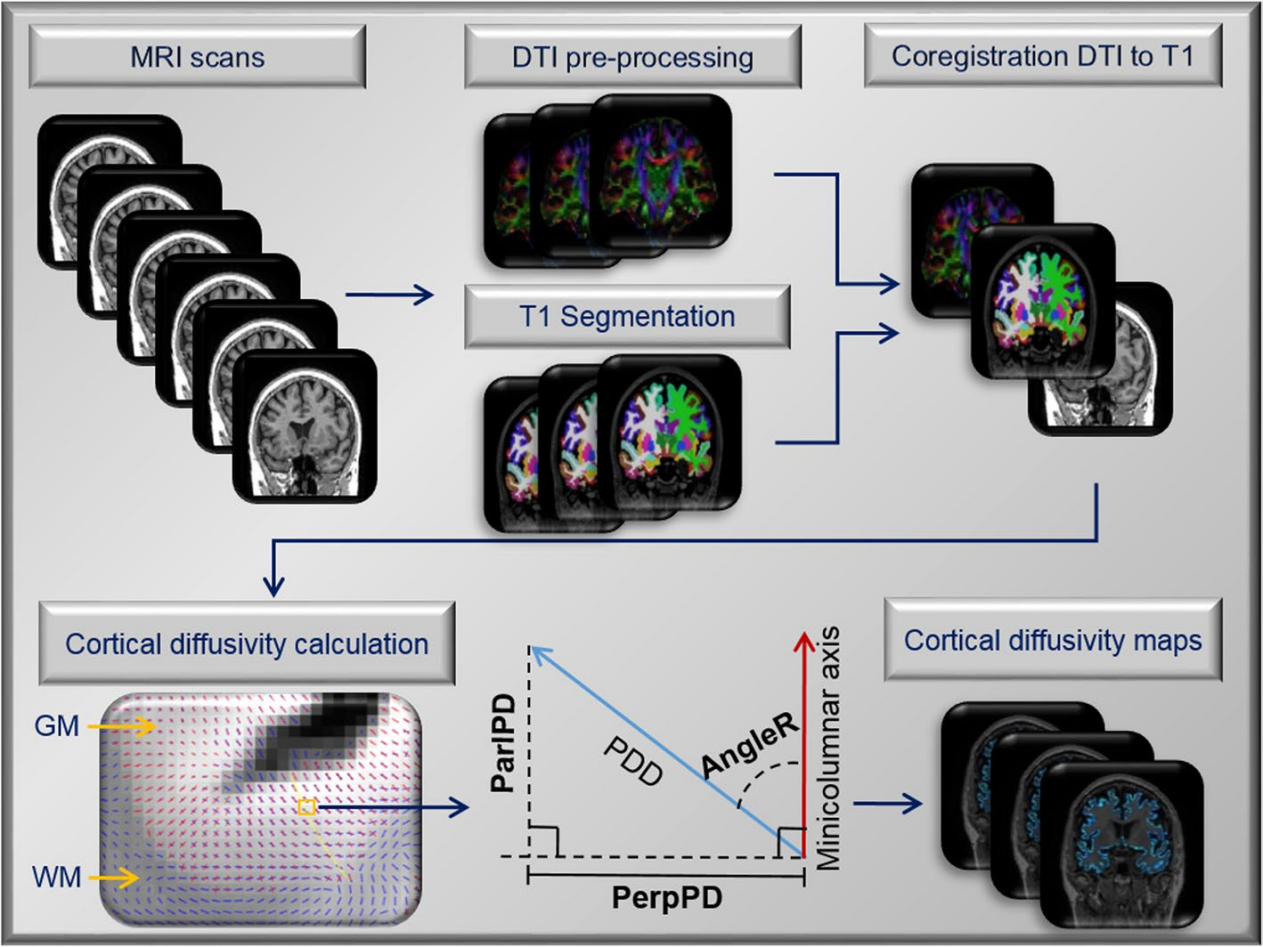
Image processing pipeline. The flow chart of the image processing and cortical diffusivity measurements

Cortical diffusivity analysis was performed using novel software scripts. The software generates cortical profiles across the cortex in a radial direction, modelling the columnar organisation within the cortex (Fig. 1) [20, 21]. Values for the diffusion tensor derived metrics were averaged along the cortical profiles, across the entire grey matter mask. The metrics calculated were MD, and three measures relating to the principal diffusion component, namely the angle between the radial minicolumn direction across the cortical layers and the principal diffusion direction (AngleR, *θ*rad); the principal diffusion component projected onto the plane perpendicular to the radial minicolumn direction across the cortex (PerpPD, (×10^–3^ mm^2^/s) and the principal diffusion component projected onto the radial minicolumn direction across the cortex (ParlPD, (×10^–3^ mm^2^/s)) [20, 21].

This set of cortical diffusivity measures have been previously correlated with amyloid and tau PET [20]. The correspondence between tau tangle pathology and cortical diffusivity has been demonstrated on a small scale by a correlation in post-mortem MRI scan data between cortical disarray measurement in the medial temporal lobe and Braak staging in histopathological microscope sections from the same subjects [32]. All the cortical values were averaged to reduce the influence of noise in the DTI scans, effectively smoothing the data, and ensuring only directionality with some local coherence would dominate, guarding against the influence of random deflections from the radial direction. Previous work has found that measures of the cyto- and myelo-architecture are relatively stable within a cortical subregion [33] indicating that it is valid to find an average value for that region.

The whole-brain DTI maps were used to extract a single value for each cortical region segmented using the recon-all pipeline of the FreeSurfer v6.0 software package (http://surfer.nmr.mgh.harvard.edu/) based on the Desikan-Killiany cortical atlas.

### Design and statistical analysis

Data were analysed using IBM SPSS Statistics version 26 (SPSS, Chicago, IL). Normality was tested using Shapiro-Wilk tests.

Analysis of variance was performed by using the multivariate General Linear Model of SPSS to compare the diagnostic group differences in cortical diffusion measurements in our cohorts, using the diagnostic group code as a fixed factor and age, sex and head motion as covariates.

All statistically significant results reported remained significant after false discovery rate correction (FDR < 0.05) [34].

### Feature reduction and classification

To assess the diagnostic group discrimination capability of the cortical diffusivity measures, in a binary (HS vs Patients) and multiclass (HS, bvFTD, svPPA, nfvPPA, PSP-RS and CBS) problem, linear discriminant analysis was used.

In the binary classification, the “best discriminator” to distinguish HS and patients was considered to be the measure with the highest accuracy statistic. Once identified in this manner, regional values of this measure were then used for the multiclass classification.

In the multiclass classification, a large number of initial features were reduced to improve the classification performance, removing irrelevant or redundant variables using principal components analysis (PCA) (SPSS Factor analysis) as a filter method. The components with a combined variance of 95% were selected for the multiclass classification.

Finally, to summarise the predictive value of each measurement, we also computed sensitivity (SENS), specificity (SPEC), positive and negative predictive values (PPV, NPV) and the positive and negative likelihood ratios (L+ and L−).

### Autopsy confirmed sample

An autopsy confirmed diagnosis was available for 17 participants, and of those, 8 cases with confirmed CBD and PSP (3 CBD and 5 PSP) were included in analyses. These subjects were included in an additional investigation to assess the diagnostic accuracy of classifying CBS and PSP-RS using regional values of the best whole brain diffusion measure and subcortical volumes. This autopsy-confirmed sub-group was used to select a set of features that were used to train a linear discriminant analysis model in the training cohort,which was then tested in the test cohort. A PCA was performed to reduce the number of variables used in the linear discriminant analysis.

## Results

### Demographics and clinical

Table 1 summarises the principal demographic and clinical characteristics of all subjects.

**Table 1:** Demographic and clinical characteristics

Entire cohort	HS = 87	bvFTD = 31	svPPA = 37	nfvPPA = 30	CBS = 39	PSP-RS = 47
**Age**^**a**^	64.3 (±6.8)	64.1 (±6.5)	64.1 (±6.7)	65.7 (±7.8)	65.3 (±6.1)	66.1 (±7.2)
**Sex M/F** ^**b**^	37/50	14/17	19/18	16/14	18/21	22/25
**Education (years)**^**a**^	17.3 (±1.9)	17.2 (±2.9)	15.3 (±3.7)	17.2 (±3.5)	16.9 (±4.1)	15.9 (±4.2)
**MMSE**^**a**^	29.5 (±0.7)	24.8 (±5.1) *	24.1 (±4.6) *	24.9 (±5.9) *	23.4 (±5.8) *	24.8 (±3.9) *
**MoCA** ^**a**^	28.1 (±1.6)	19.1 (±4.2) *	17.6 (±6.9) *	20.5 (±6.3) *	19.1 (±6.4) *	21.1 (±4.3) *
**CDR global %**	0=100 0.5=0 1=0 2=0 3=0	0=3.2 0.5=29.1 1=41.9 2=25.8 3=0	0=0 0.5=51.4 1=29.7 2=18.9 3=0	0=10 0.5=43.3 1=30 2=16.7 3=0	0=2.9 0.5=48.6 1=34.3 2=11.3 3=2.9	0=9.1 0.5=45.4 1=36.4 2=9.1 3=0
**CDR-SB**^**a**^	0	5.4 (±3.1) *	3.65 (±2.5) *	2.9 (±1.6)	3.8 (±3.2) *	4.1 (±3.1) *
**UPDRS**^**a**^	0.1 (±0.5)	2.7 (±3.1) *	4.6 (±5.5) *	8.1 (±9.6) *	33.3 (±15.1) *#	35.0 (±18.2) *#
**Training cohort**	**HS = 58**	**bvFTD = 15**	**svPPA = 21**	**nfvPPA = 17**	**CBS = 24**	**PSP-RS = 32**
**Age**^**a**^	64.1 (7.1)	64.5 (6.4)	64.5 (6.4)	65.5 (7.1)	66.2 (6.2)	66.5 (6.9)
**Sex M/F**^**b**^	24/34	7/8	11/10	9/8	11/13	15/17
**Education (years)**^**a**^	17.3 (1.8)	17.4 (3.5)	15.7 (3.9)	16.7 (4.3)	17.5 (4.3)	14.9 (4.2)
**MMSE**^**a**^	29.4 (0.8)	24.2 (3.9) *	23.2 (4.6) *	25.5 (4.6) *	23.8 (5.7) *	25.1 (3.5) *
**MoCA**^**a**^	27.9 (1.7)	18.7 (3.5) *	18.5 (5.1) *	19.3 (7.2) *	19.3 (6.1) *	21.6 (2.9) *
**CDR global %**	0=100 0.5=0 1=0 2=0 3=0	0=0 0.5=26.7 1=46.7 2=26.7 3=0	0=0 0.5=52.4 1=28.6 2=19 3=0	0=5.9 0.5=47.1 1=29.4 2=17.6 3=0	0=5 0.5=50 1=35 2=10 3=0	0=10.3 0.5=48.3 1=34.5 2=6.9 3=0
**CDR-SB**^**a**^	0	5.5 (±3.5) *	4 (±2.2) *	2.8 (±1.2)	3.4 (±2.1) *	4.2 (±3.1) *
**UPDRS**^**a**^	0 (0)	2.7 (±3.2) *	4.1 (±4.8) *	7.2 (±8.1) *	33.1 (±11.1) *#	35.3 (±19.8) *#
**Test cohort**	**HS = 29**	**bvFTD = 16**	**svPPA = 16**	**nfvPPA = 13**	**CBS = 15**	**PSP-RS = 15**
**Age**^**a**^	64.7 (±5.6)	63.6 (±6.4)	63.6 (±7.2)	66.2 (±6.8)	64.4 (±6.3)	65.6 (±8.3)
**Sex M/F**^**b**^	13/16	7/9	8/8	7/6	7/8	7/8
**Education (years)**^**a**^	17.4 (±1.8)	17.0 (±2.7)	14.8 (±3.3)	18.2 (±1.7)	15.5 (±3.1)	17.6 (±3.5)
**MMSE**^**a**^	29.5 (±0.6)	25.2 (±5.9) *	25.4 (±4.4) *	23.6 (±8.3) *	22.7 (±6.4) *	24.2 (±4.9) *
**MoCA**^**a**^	28.5 (±1.4)	19.4 (±4.9) *	16.4 (±9.1) *	22.4 (±4.1) *	18.4 (±7.8) *	19.6 (±6.1) *
**CDR global %**	0=100 0.5=0 1=0 2=0 3=0	0=6.25 0.5=31.25 1=37.5 2=25 3=0	0=0 0.5=50 1=31.3 2=18.7 3=0	0=15.4 0.5=38.5 1=30.7 2=15.4 3=0	0=0 0.5=46.6 1=33.3 2=13.3 3=6.7	0=6.7 0.5=40 1=40 2=13.3 3=0
**CDR-SB**^**a**^	0	5.3 (±3.9) *	3.3 (±2.7) *	2.8 (±2.4)	4.5 (±4.2) *	3.8 (±2.8) *
**UPDRS** ^**a**^	0.4 (±0.9)	2.7 (±3.1) *	5.3 (±6.39) *	9.7 (±10.2) *	33.7 (±18.2) *#	34.5 (±15.5) *#

*MMSE* Mini-Mental State Examination; *MoCA* Montreal Cognitive Assessment; *CDR* Clinical Dementia Rating scale, global score; *CDR-SB* Clinical Dementia Rating scale, sum of boxes; *UPDRS* Unified Parkinson’s Disease Rating Scale

^a^ANOVA

^b^Chi-square

*P < 0.05 patient group versus HS, FDR corrected; ^#^*P* < 0.05 patient group versus bvFTD, svPPA, nfvPPA, FDR corrected

No differences in age, sex and education were detected. As expected, all patient groups were different with respect to the control group in MMSE (*p* < 0.001), MoCA (*p* < 0.001), CDR (*p* < 0.001) and UPDRS (*p* < 0.001). However, CBS and PSP-RS groups obtained significantly higher UPDRS scores compared to the other patient groups (bvFTD, svPPA and nfvPPA).

The same differences were found in the subsets when splitting the cohort into a Training and Test cohort.

### Cortical assessment

In both cohorts, all patients combined together as a group were significantly different from the control group in all measures considered (AngleR, PerpPD, ParlPD, MD, Cortical Thickness and GM_fr). In the training cohort the GLM revealed significant effects of diagnostic group on AngleR (*F*_4,156_ = 19.07, *p* = 0.000, *η*^2^_*p*_=0.109), PerpPD (*F*_4,156_ = 135.96, *p* = 0.000, *η*^2^_*p*_=0.466), MD (*F*_4,156_ = 61,04, *p* = 0.000, *η*^2^_*p*_=0.281) ParlPD (*F*_4,156_ = 94.37, *p* = 0.000, *η*^2^_*p*_=0.377), Cortical Thickness (*F*_4,156_ = 54.18, *p* = 0.000, *η*^2^_*p*_=0.258) and GM_fr (*F*_4,156_ = 47.67, *p* = 0.000, *η*^2^_*p*_=0.234). No significant effects of age, sex or head motion on diffusion values were detected (FDR < 0.05; 5 tests).

Similar to the training cohort, the GLM in the test cohort showed significant effects of diagnostic group on AngleR (*F*_4,96_ = 27.14, *p* = 0.000, *η*^2^_*p*_=0.258), PerpPD (*F*_4,96_ = 35.65, *p* = 0.000, *η*^2^_*p*_=0.314), MD (*F*_4,96_ = 27.57, *p* = 0.000, *η*^2^_*p*_=0.261), ParlPD (*F*_4,96_ = 9.13, *p* = 0.003, *η*^2^_*p*_=0.105), Cortical Thickness (*F*_4,96_ = 13.15, *p* = 0.001, *η*^2^_*p*_=0.144), and GM_fr (*F*_4,96_ = 15.92, *p* = 0.000, *η*^2^_*p*_=0.179). No significant effects of age, sex or head motion on diffusion values were detected (FDR < 0.05; 5 tests).

In each cohort, all groups were compared with each other for all variables and the results are summarised in Table 2.

**Table 2:** Cortical investigation

Training cohort	AngleR	PerpPD	ParlPD	MD	Cortical thickness	GM_fr
**HS vs SV**	*η*^2^_*p*_=0.159*	*η*^2^_*p*_ =0.374*	*η*^2^_*p*_ =0.166*	*η*^2^_*p*_ =0.168*	*η*^2^_*p*_ =0.279*	*η*^2^_*p*_ =0.146*
**HS vs BV**	*η*^2^_*p*_ =0.116*	*η*^2^_*p*_ =0.537*	*η*^2^_*p*_ =0.426*	*η*^2^_*p*_ =0.353*	*η*^2^p =0.327*	*η*^2^_*p*_ =0334*
**HS vs PNFA**	*η*^2^_*p*_ *=0.064*	*η*^2^_*p*_ =0.314*	*η*^2^_*p*_ =0.173*	*η*^2^_*p*_ =0115*	*η*^2^*p =0.055*	*η*^2^_*p*_ *=0.040*
**HS vs CBS**	*η*^2^_*p*_ *=0.021*	*η*^2^_*p*_ =0.338*	*η*^2^_*p*_ =0.220*	*η*^2^_*p*_ =0.116*	*η*^2^_*p*_ *=0.061*	*η*^2^_*p*_ *=0.049*
**HS vs PSP-RS**	*η*^2^_*p*_ *=0.004*	*η*^2^_*p*_ =0.126*	*η*^2^_*p*_ =0.123*	*η*^2^_*p*_ *=0.060*	*η*^2^_*p*_ *=0078*	*η*^2^_*p*_ *=0.087*
**SV vs BV**	*η*^2^_*p*_ *=0.010*	*η*^2^_*p*_ *=0.020*	*η*^2^_*p*_ *=0.064*	*η*^2^_*p*_ *=0.027*	*η*^2^_*p*_ *=0.000*	*η*^2^_*p*_ *=0.030*
**SV vs PNFA**	*η*^2^_*p*_ *=0.021*	*η*^2^_*p*_ *=0.009*	*η*^2^_*p*_ *=0.000*	*η*^2^_*p*_ *=0.006*	*η*^2^_*p*_ *=0.088*	*η*^2^_*p*_ *=0.028*
**SV vs CBS**	*η*^2^_*p*_ *=0.065*	*η*^2^_*p*_ *=0.019*	*η*^2^_*p*_ *=0.000*	*η*^2^_*p*_ *=0.014*	*η*^2^_*p*_ =0.107*	*η*^2^_*p*_ *=0.033*
**SV vs PSP-RS**	*η*^2^_*p*_ =0.108*	*η*^2^_*p*_ =0.158*	*η*^2^_*p*_ *=0.019*	*η*^2^_*p*_ *=0.050*	*η*^2^_*p*_ =0.114*	*η*^2^_*p*_ *=0.022*
**BV vs PNFA**	*η*^2^_*p*_ *=0.003*	*η*^2^_*p*_ *=0.059*	*η*^2^_*p*_ *=0.067*	*η*^2^_*p*_ *=0.062*	*η*^2^_*p*_=0.094*	*η*^2^_*p*_ =0.116*
**BV vs CBS**	*η*^2^_*p*_ *=0.031*	*η*^2^_*p*_ =0.098*	*η*^2^_*p*_ *=0.073*	*η*^2^_*p*_ *=0.090*	*η*^2^_*p*_ =0.119*	*η*^2^_*p*_ *=0.116*
**BV vs PSP-RS**	*η*^2^_*p*_ *=0.065*	*η*^2^_*p*_ =0.291*	*η*^2^_*p*_ =0.173*	*η*^2^_*p*_ =0.165*	*η*^2^_*p*_ =0.125*	*η*^2^_*p*_ *=0.120*
**PNFA vs CBS**	*η*^2^_*p*_ *=0.013*	*η*^2^_*p*_ *=0.001*	*η*^2^_*p*_ *=0.000*	*η*^2^_*p*_ *=0.001*	*η*^2^_*p*_ *=0.000*	*η*^2^_*p*_ *=0.000*
**PNFA vs PSP-RS**	*η*^2^_*p*_ *=0.037*	*η*^2^_*p*_ =0.109*	*η*^2^_*p*_ *=0.021*	*η*^2^_*p*_ *=0.021*	*η*^2^_*p*_ *=0.000*	*η*^2^_*p*_ *=0.002*
**CBS vs PSP-RS**	*η*^2^_*p*_ *=0.006*	*η*^2^_*p*_ =0.100*	*η*^2^_*p*_ *=0.028*	*η*^2^_*p*_ *=0.014*	*η*^2^_*p*_ *=0.000*	*η*^2^_*p*_ *=0.002*
**Test cohort**	**AngleR**	**PerpPD**	**ParlPD**	**MD**	**Cortical thickness**	**GM_fr**
**HS vs SV**	*η*^2^_*p*_ =0.293*	*η*^2^_*p*_ =0.340*	*η*^2^_*p*_ =0.100*	*η*^2^_*p*_ =0.188*	*η*^2^_*p*_ =0.228*	*η*^2^_*p*_ =0.141*
**HS vs BV**	*η*^2^_*p*_ =0.229*	*η*^2^_*p*_ =0.362*	*η*^2^_*p*_ =0.170*	*η*^2^_*p*_ =0.264*	*η*^2^_*p*_ =0.257*	*η*^2^_*p*_ =0.205*
**HS vs PNFA**	*η*^2^_*p*_ *=0.072*	*η*^2^_*p*_ =0.247*	*η*^2^_*p*_ =0.108*	*η*^2^_*p*_ =0.168*	*η*^2^_*p*_ *=0.045*	*η*^2^_*p*_ *=0.043*
**HS vs CBS**	*η*^2^_*p*_ =0.118*	*η*^2^_*p*_ =0.248*	*η*^2^_*p*_ =0.102*	*η*^2^_*p*_ =0.215*	*η*^2^_*p*_ *=0.058*	*η*^2^_*p*_ *=0.086*
**HS vs PSP-RS**	*η*^2^_*p*_ =0.101*	*η*^2^_*p*_ =0.109*	*η*^2^_*p*_ *=0.007*	*η*^2^_*p*_ *=0.033*	*η*^2^_*p*_ *=0.000*	*η*^2^_*p*_ *=0.039*
**SV vs BV**	*η*^2^_*p*_ *=0.002*	*η*^2^_*p*_ *=0.010*	*η*^2^_*p*_ *=0.023*	*η*^2^_*p*_ *=0.027*	*η*^2^_*p*_ *=0.009*	*η*^2^_*p*_ *=0.020*
**SV vs PNFA**	*η*^2^_*p*_ *=0.046*	*η*^2^_*p*_ *=0.001*	*η*^2^_*p*_ *=0.006*	*η*^2^_*p*_ *=0.004*	*η*^2^_*p*_ *=0.039*	*η*^2^_*p*_ *=0.010*
**SV vs CBS**	*η*^2^_*p*_ *=0.067*	*η*^2^_*p*_ *=0.019*	*η*^2^_*p*_ *=0.000*	*η*^2^_*p*_ *=0.002*	*η*^2^_*p*_ *=0.066*	*η*^2^_*p*_ *=0.004*
**SV vs PSP-RS**	*η*^2^_*p*_ *=0.042*	*η*^2^_*p*_ =0.180*	*η*^2^_*p*_ =0.113*	*η*^2^_*p*_ *=0.046*	*η*^2^_*p*_ =0.151*	*η*^2^_*p*_ *=0.019*
**BV vs PNFA**	*η*^2^_*p*_ *=0.030*	*η*^2^_*p*_ *=0.005*	*η*^2^_*p*_ *=0.002*	*η*^2^_*p*_ *=0.004*	*η*^2^_*p*_ *=0.065*	*η*^2^_*p*_ *=0.040*
**BV vs CBS**	*η*^2^_*p*_ *=0.041*	*η*^2^_*p*_ *=0.048*	*η*^2^_*p*_ *=0.029*	*η*^2^_*p*_ *=0.013*	*η*^2^_*p*_ =0.102*	*η*^2^_*p*_ *=0.037*
**BV vs PSP-RS**	*η*^2^_*p*_ *=0.024*	*η*^2^_*p*_ =0.210*	*η*^2^_*p*_ =0.171*	*η*^2^_*p*_ =0.102*	*η*^2^_*p*_ =0.179*	*η*^2^_*p*_ *=0.057*
**PNFA vs CBS**	*η*^2^_*p*_ *=0.001*	*η*^2^_*p*_ *=0.012*	*η*^2^_*p*_ *=0.011*	*η*^2^_*p*_ *=0.000*	*η*^2^_*p*_ *=0.000*	*η*^2^_*p*_ *=0.002*
**PNFA vs PSP-RS**	*η*^2^_*p*_ *=0.001*	*η*^2^_*p*_ =0.146*	*η*^2^_*p*_ =0.131*	*η*^2^_*p*_ *=0.061*	*η*^2^_*p*_ *=0.030*	*η*^2^_*p*_ *=0.000*
**CBS vs PSP-RS**	*η*^2^_*p*_ *=0.000*	*η*^2^_*p*_ =0.114*	*η*^2^_*p*_ =0.102*	*η*^2^_*p*_ *=0.065*	*η*^2^_*p*_ *=0.033*	*η*^2^_*p*_ *=0.006*

General Linear Model (GLM) post hoc. *η*^2^_*p*_ = partial eta-squared. All values reported remained statistically significant after false discovery rate correction (FDR < 0.05)

### Binary classification

The results of the linear discriminant analysis in the binary problem (HS vs all patients) are summarised in Table 3. The whole brain measures analysed together provided an accuracy of 80%.

**Table 3:** Binary classification HS vs all patients

Test cohort	Accuracy %	SENS %	SPEC %	PPV %	NPV %	LR+	LR-
**All measures**	80	81.9	75.9	89.4	62.9	3.4	0.24
**PerpPD**	**86**	**83.1**	**93.1**	**96.7**	**69.2**	**12.1**	**0.18**
**ParlPD**	80	78.9	82.8	91.8	61.5	4.6	0.26
**AngleR**	77	74.6	82.8	91.4	57.1	4.3	0.31
**MD**	75	73.2	79.3	89.7	54.8	3.5	0.34
**Cortical Thickness**	72	70.8	75.9	87.9	51.2	2.9	0.38
**Cortical GMfr**	71	69.0	75.9	87.5	50.0	2.9	0.41

Table 3 shows the accuracies for binary classification using whole brain diffusion and volumetric measures. Abbreviations: *SENS* sensitivity, *SPEC* specificity, *PPV* positive predictive values, *NPV* negative predictive value, *LR+* positive likelihood ratio, *LR−* negative likelihood ratio

Concerning the single feature performances (mean whole brain values) in the test cohort (using the model derived from the training cohort), PerpPD was the feature with the highest accuracy (86%) followed by ParlPD (80%), AngleR (77%), MD (75%), Cortical thickness (72%) and GM_fr (71%). The single measure with the highest accuracy (PerpPD) was then extracted at the regional level to investigate the differences among FTLD syndromes in the multiclass classification.

### Feature selection and multiclass classification

**Fig. 2:**
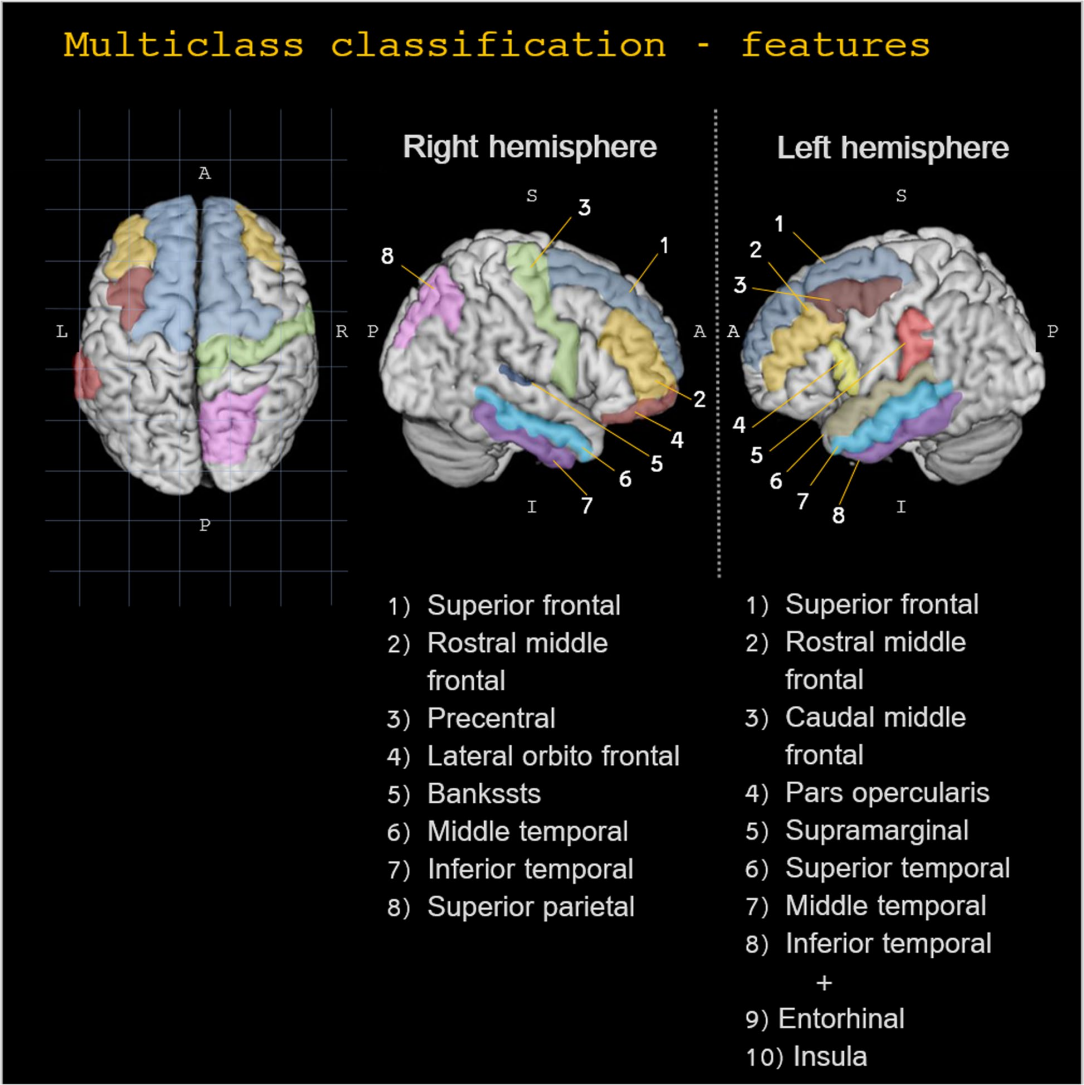
Multiclass classification features. The regional PerpPD values used in multiclass classification. The features could be roughly grouped into three main patterns: frontal, temporal and parietal

A collection of 19 regional PerpPD values (Table 4 and Fig. 2) were selected using PCA in the training cohort. These features were used in the training cohort to create a model that was then tested on the Test cohort.

**Table 4:** Multi-class classification features selected

	Features	Lobe
***Left Hemisphere***	PerpPD inferior temporal L	Temporal
	PerpPD middle temporal L	Temporal
	PerpPD insula L	Temporal
	PerpPD entorhinal L	Temporal
	PerpPD superior temporal L	Temporal
	PerpPD caudal middle frontal L	Frontal
	PerpPD rostral middle frontal L	Frontal
	PerpPD superior frontal L	Frontal
	PerpPD pars opercularis L	Frontal
	PerpPD supramarginal L	Parietal
	PerpPD bankssts R	Temporal
	PerpPD inferior temporal R	Temporal
	PerpPD middle temporal R	Temporal
	PerpPD rostral middle frontal R	Frontal
***Right Hemisphere***	PerpPD superior frontal R	Frontal
	PerpPD precentral R	Frontal
	PerpPD lateral orbito frontal R	Frontal
	PerpPD superior parietal R	Parietal
***Whole Brain***	PerpPD	

Table 4 shows the PerpPD values selected by PCA and used in the multiclass classification

The linear discriminant analysis results showed that the trained model was able to differentiate among FTLD syndromes with an accuracy of 85.6%. The confusion matrix is shown in Table 5.

**Table 5:**
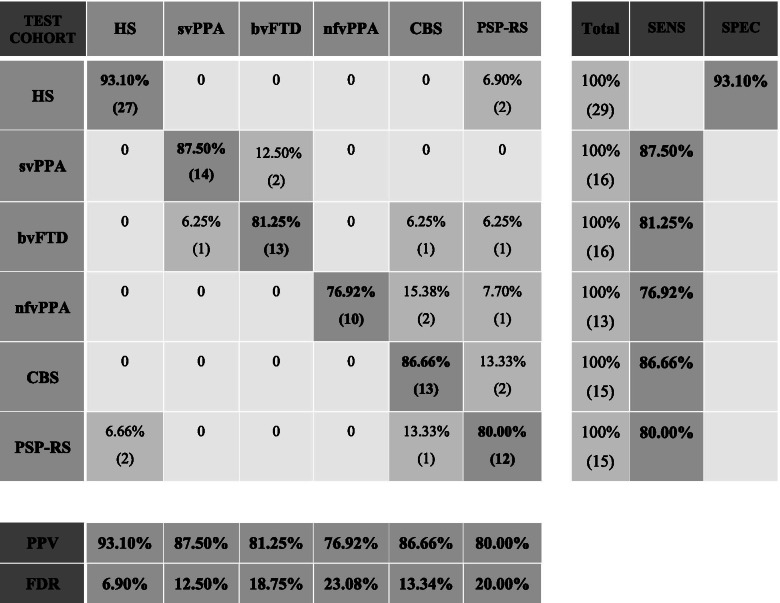
Multiclass confusion matrix

Table 5 shows the multiclass confusion matrix obtained using the PerpPD values

**Fig. 3:**
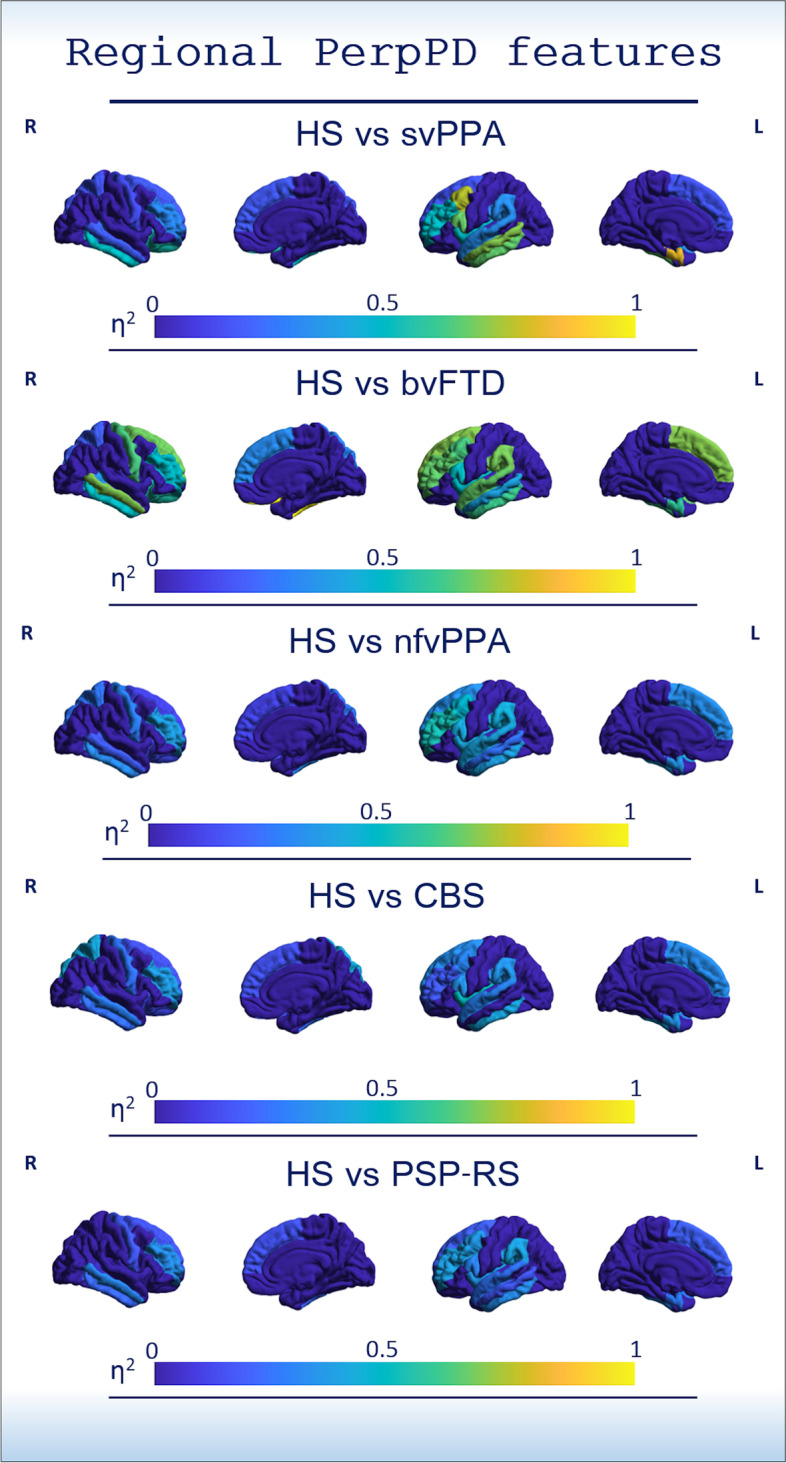
The colour maps indicate the effect size. Effect-size maps (partial eta squared *η*^2^_*p*_) were created for each FTLD syndrome compared to HS, using the regional PerpPD values included as a feature in the multiclass challenge

To describe differences in the regional PerpPD features, effect-size maps were created testing each patient group for greater regional PerpPD than the control group (Fig. 3).

### Autopsy confirmation sample

The two autopsy-confirmed groups of cases (CBD and PSP) were not significantly different for age or sex. Comparing structural MRI measures, no differences in cortical GM volume fraction, WM volume fraction or cortical thickness were detected, while the two groups showed a significant difference in Brainstem volume fraction (*F*_1,7_ = 31.95, *p*=0.001, *η*^*2*^_*p*_*=*0.820). The features selected by PCA of the autopsy group and used in the linear discriminant analysis were left and right PerpPD caudal anterior cingulate cortex, left and right PerpPD superior parietal cortex, right PerpPD postcentral cortex, right PerpPD supramarginal cortex and brainstem volume fraction.

**Fig. 4:**
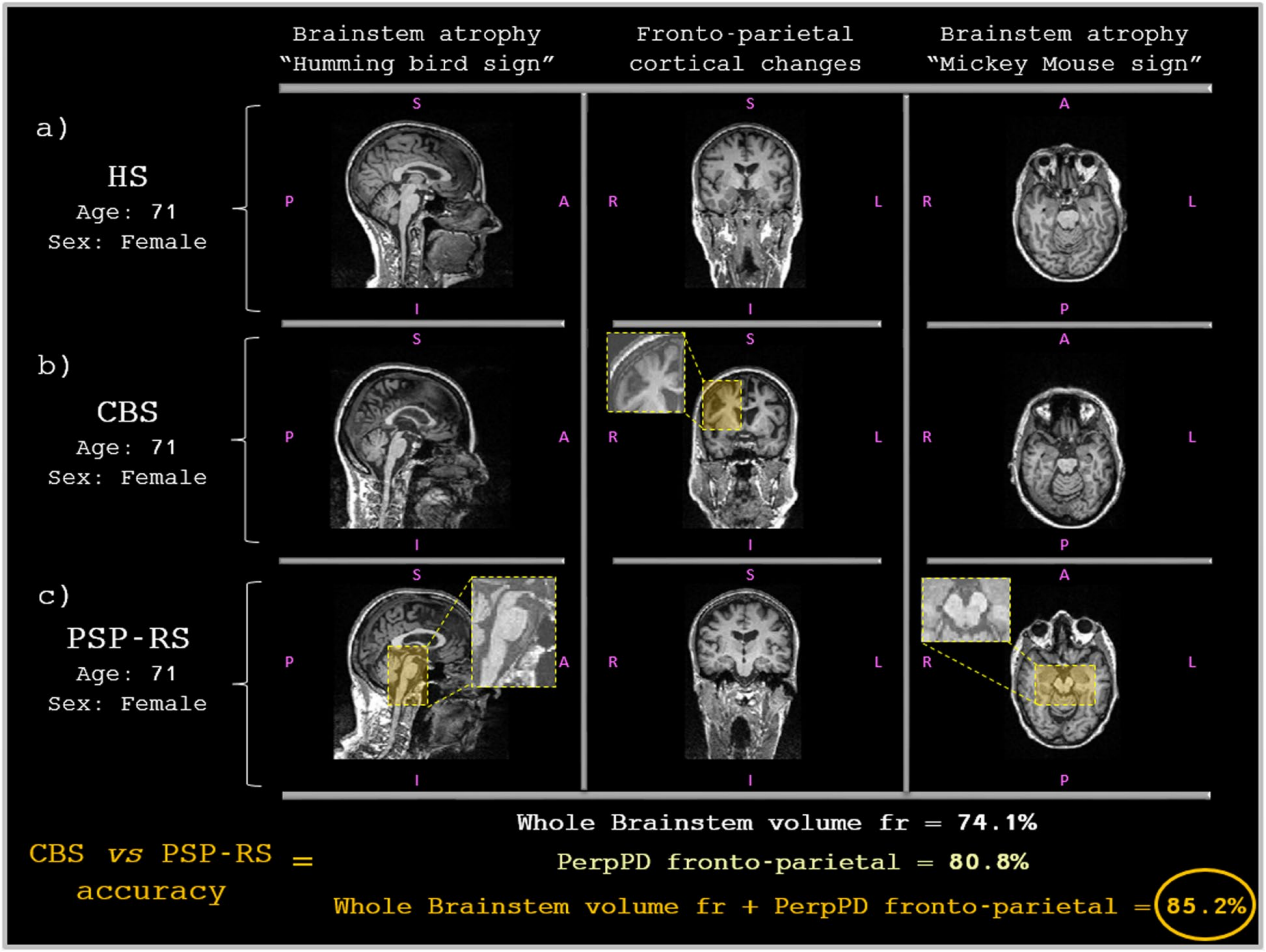
CBS vs PSP-RS classification. Comparison of three age- and sex-matched individuals, showing the features used in the binary classification of CBS vs PSP-RS

These features, selected in the autopsy confirmed cohort, were used together to train a model using the training cohort, which was then tested in the test cohort, classifying CBS and PSP-RS with an accuracy of 85.2%. Used separately, the six diffusion values of cortical diffusivity, without brainstem, obtained an accuracy of 80.8% while the whole brainstem volume fraction obtained an accuracy of 74.1% (see Table 6 and Fig. 4).

**Table 6:** CBS vs PSP-RS binary classification

Test cohort	Accuracy %	CBS Accuracy %	PSP-RS Accuracy %
**Regional PerpPD selected features**			
**L and R caudal anterior cingulate PerpPD** **L and R superior parietal PerpPD** **R postcentral PerpPD** **R supramarginal PerpPD**	**80.8**	**91.7**	**71.4**
**Brainstem substructures**			
**Whole brainstem volume fraction**	**74.1**	**75.0**	**73.3**
**Midbrain volume fraction**	**70.4**	**83.3**	**60**
**Medulla volume fraction**	**66.7**	**83.3**	**53.3**
**Pons volume fraction**	**70.4**	**66.7**	**73.3**
**Superior cerebellar peduncle volume fraction**	**66.7**	**91.7**	**46.7**
**Regional PerpPD selected features + whole brainstem**			
**L and R caudal anterior cingulate PerpPD** **L and R superior parietal PerpPD** **R postcentral PerpPD** **R supramarginal PerpPD** **+** **Whole brainstem volume fraction**	**85.2**	**91.7**	**80.0**

Table 6 shows the classification accuracy of PerpPD values and brainstem substructures volume fractions

Comparing the two groups, *T* tests showed significantly higher PerpPD values (left and right superior parietal PerpPD, right postcentral PerpPD) in CBS, while PSP-RS showed a lower brainstem volume fraction (Table 7).

**Table 7:** CBS vs PSP-RS features comparison

Features selected	CBS vs PSP-RS	***p*** value
**Left caudal anterior cingulate PerpPD**	CBS < PSP-RS	n.s
**Right caudal anterior cingulate PerpPD**	CBS < PSP-RS	n.s
**Left superior parietal PerpPD**	CBS > PSP-RS	*p*=0.000, *η*^2^_*p*_*=*0.423
**Right superior parietal PerpPD**	CBS > PSP-RS	*p*=0.001, *η*^2^_*p*_*=*0.351
**Right postcentral PerpPD**	CBS > PSP-RS	*p*=0.008, *η*^2^_*p*_*=*0.250
**Right supramarginal PerpPD**	CBS > PSP-RS	n.s
**Brainstem volume fraction**	CBS > PSP-RS	*p*=0.008, *η*^*2*^_*p*_*=*0.322

Table 7 *η*^2^_*p*_*=*partial eta-squared. All *p* values reported remained statistically significant after false discovery rate correction (FDR < 0.05; 7 tests)

## Discussion

The main goal of the present study was to investigate the cortical microstructural signature in FTLD-related syndromes, using novel cortical diffusion measures and testing their diagnostic power. Three different challenges were investigated: a binary classification (controls vs all patients), a multiclass classification (HS vs bvFTD vs svPPA vs nfvPPA vs CBS vs PSP-RS) and an additional binary classification to differentiate CBS and PSP-RS using features selected in an autopsy confirmed subcohort (CBD vs PSP).

### Binary classification

In the binary classification (controls vs all patients), 3 novel cortical diffusion measures (AngleR, PerpPD and ParlPD), a conventional diffusion measures (MD), cortical thickness and the whole brain cortical grey matter fraction were used. The diagnostic accuracy was calculated using these features together and one by one. Grouped together these features were capable of differentiating controls and patients with an accuracy of 80% while the single feature with the highest accuracy was the whole brain PerpPD (86%). Although, as illustrated by the literature, all these FTLD clinical syndromes show different patterns of cortical damage [35, 36], the results revealed that the whole brain cortical diffusion measures can distinguish between controls and patients with a good level of diagnostic accuracy.

Previous studies showed that cortical diffusion measures are sensitive to the cortical changes in FTLD [22, 27, 28]. These measures may act as surrogate measures of cytoarchitectural features such as minicolumn structure and cell distribution and may also be sensitive to changes in organisation related to neuropathology including pathological protein accumulation [20, 21]. In general, the distance travelled by water molecules during the diffusion scan is of the order of a few microns. As demonstrated in white matter, the microstructural features contributing to the signal are complex but are likely to include myelin sheaths around axons and the microtubules that are aligned within the neurites. In histopathological assessment, Giannini et al. [37] reported different anatomical distributions of pathology in both white matter and grey matter in FTLD-Tau compared to FTLD-TDP. It is not yet certain which elements of either pathological form contributes most strongly to the signals seen in cortical diffusivity assessment. We speculate that PerpPD may be sensitive to pathology of tau, which normally lends structural integrity to the microtubules, and is therefore associated with disruption of the microtubules within neurite bundles that are aligned to the minicolumn.

### Multiclass classification

In the multiclass challenge, the 19 regional PerpPD values selected by PCA (Table 4 and Fig. 2) enabled a classification accuracy of 86%. More specifically, the PerpPD whole-brain values plus 18 out of 68 regional PerpPD values were used. These regions correspond to those mainly involved in FTD subtypes and CBS [38, 39]. As shown by the confusion matrix, the linear discriminant analysis showed that the features included had a high specificity in classifying the HS (93.1%). Regarding the patient groups, the confusion matrix showed that the classification accuracy ranged between 76.9 and 87.5%.

Although the underlying neuropathological mechanisms in the diagnostic groups considered in this classification could be different [8], the findings confirmed that the diffusion measures are sensitive to the cortical changes caused by FTLD-related neurodegeneration. In addition, the topographical distribution of cortical alterations may be useful to detect cortical patterns of disease-related neurodegeneration. This could be used to help clinicians in differential diagnosis, reducing misclassification.

These cortical diffusion changes, reflecting progressing microstructural degeneration appear to be present in the absence of substantial atrophy and may precede detectable macroscopic atrophy. This has the potential to help clinicians in diagnosis, particularly in the early stages of the disease, when the pattern of atrophy is not clear.

As shown previously [22], cortical diffusivity measures might be more sensitive than cortical thickness to detect the earliest disease-related cortical changes in bvFTD.

There may be a benefit particularly for differentiating bvFTD in the early stages from primary psychiatric conditions. When disinhibition is clear, the overlap between bipolar disorder or schizophrenia may not be too difficult to disambiguate, based on the age and clinical history of the patient. However, bvFTD can onset at a relatively young age, for example, in the 50s. In the prodromal phase, some of the more subtle symptoms can be difficult to differentiate from other conditions; for example, apathy may manifest like depression or irritability may appear like anxiety.

### CBS vs PSP-RS: autopsy confirmed

The discriminant analysis performed using the features selected in the autopsy-confirmed subcohort, showed, in the test cohort, a classification accuracy of 85.2% using 6 fronto-parietal PerpPD values and the whole brainstem volume fraction.

More specifically, the group comparisons (Table 7) showed cortical microstructural differences in the parietal regions (bilateral superior parietal and post central cortex) with higher values in CBS individuals, while the PSP-RS group showed lower brain stem volume fraction.

The wider cortical distribution of cortical DTI changes in CBS than PSP-RS subjects and the bigger volume loss of brainstem in PSP-RS subjects is largely consistent with histopathological findings described in the literature [36, 39].

The two groups were not significantly different in the cortical grey matter fraction suggesting that the cortical diffusion changes prominent in CBS are not dependent on macroscopic cortical changes.

Microscopically, in CBD the cortical areas show neuronal loss with astrocytosis [40], often most severe in the superficial cortical laminae associated with superficial spongiosis [41]. Severely affected cortex shows loss of laminar architecture, transcortical microvacuolation and marked astrocytosis most frequently in cortical layers III, V and VI [41]. A large part of these layers (III and V) is normally characterised by the most well-defined minicolumn structure and, therefore, changes of their geometrical properties, sensitive to pathology in those layers, could contribute to the difference in PerpPD values.

In addition, the higher frontal and parietal PerpPD values found in CBS individuals may be related to the effects of astrocytic plaque pathology. These plaques are frequent in posterior frontal and parietal cortices and can be found throughout the striatum [41].

Conversely, histopathologically, PSP is characterised by less cortical damage and neuronal loss, gliosis, and abundant neurofibrillary tangles in the basal ganglia, midbrain and brainstem [41, 42]. The group comparisons are consistent with these histopathological changes, revealing significant differences in brain stem fraction where the PSP-RS group showed a lower brainstem fraction compared to the CBS group.

Although CBS and PSP-RS have different clinical phenotypes, they can have overlapping pathological underpinnings, making differential diagnosis difficult. Combining volumetric and cortical diffusion measures may improve the diagnosis, reducing misclassification and allowing clearer, more personalised diagnosis.

### Limitations

First, after splitting the cohort into a training cohort and a test cohort, the sample size of some remaining diagnostic groups was limited. This is acknowledged throughout the interpretation of the findings, which we expect to be reproducible in future research on a larger cohort.

Another avenue for extending this work would be to explore the relationship with other biomarker measurements such as tau protein quantification using CSF or PET markers which were not available for this study.

It must be acknowledged that the spatial resolution of DTI is limited and although it is meaningful to average values within cortical regions in the Desikan-Killiany atlas due to the relative cytoarchitectural homogeneity of cortical regions, the spatial resolution must be considered when interpreting the neuropathological correspondence to DTI changes in the cortex.

Furthermore, out of the 271 subjects included in the study, 17 had autopsy confirmation, and 8 were used to confirm the underlying neuropathology. It should be noted that the presence of Alzheimer’s disease and its contribution to pathology, either as the primary neuropathology or as a comorbidity, cannot be clarified until autopsy. Post-mortem examination had not, therefore, been conducted or made available for the majority of subjects in this study and therefore cannot be conclusively ruled out for those subjects.

Additional histology studies in the future will be helpful to better characterise the intracortical diffusivity signature for each diagnostic group.

## Conclusion

These results, taken together, suggest that cortical diffusivity changes show promise as a non-invasive measure of neurodegeneration in FTLD syndromes and could be further developed to support the diagnosis of neurodegenerative diseases. Although the clinical presentations of FTLD studied here are included in the same broad neuropathological spectrum, they show different patterns of cortical neurodegeneration that are detectable using cortical diffusivity measures. Further investigations could characterise the microstructural cortical signature in other FTLD syndromes as well.

## Data Availability

The data supporting the conclusions of this article are available from the corresponding author on reasonable request. The datasets analysed during the current study are available in [LONI Image Data Archive] at https://ida.loni.usc.edu/login.jsp.

## Abbreviations

FTD: Frontotemporal dementia
BV: Behavioural variant
SV: Semantic variant
PNFA: Progressive non-fluent aphasia
CBD: Corticobasal degeneration
CBD: Corticobasal syndrome
PSP-RS: Progressive supranuclear palsy Richardson’s syndrome
DTI: Diffusion tensor imaging
AngleR: Angle between the principal diffusion direction and the radial minicolumn direction across the cortex
PerpPD: The component of the principal diffusion vector that was perpendicular to the radial minicolumn direction across the cortex
ParlPD: the component of the principal diffusion vector that was parallel to the radial minicolumn direction across the cortex
GM: Grey matter
MD: Mean diffusivity
